# Enduring Fluoride Health Hazard for the Vesuvius Area Population: The Case of AD 79 *Herculaneum*

**DOI:** 10.1371/journal.pone.0021085

**Published:** 2011-06-16

**Authors:** Pierpaolo Petrone, Michele Giordano, Stefano Giustino, Fabio M. Guarino

**Affiliations:** 1 Museo di Antropologia, Centro Musei delle Scienze Naturali, Università degli Studi di Napoli Federico II, Naples, Italy; 2 Istituto per i Materiali Compositi e Biomedici, Consiglio Nazionale delle Ricerche IMCB-CNR, Portici, Italy; 3 Dipartimento di Biologia Strutturale e Funzionale, Università degli Studi di Napoli Federico II, Complesso Monte S. Angelo, Naples, Italy; University of Delaware, United States of America

## Abstract

**Background:**

The study of ancient skeletal pathologies can be adopted as a key tool in assessing and tracing several diseases from past to present times. Skeletal fluorosis, a chronic metabolic bone and joint disease causing excessive ossification and joint ankylosis, has been only rarely considered in differential diagnoses of palaeopathological lesions. Even today its early stages are misdiagnosed in endemic areas.

**Methodology/Principal Findings:**

Endemic fluorosis induced by high concentrations of fluoride in water and soils is a major health problem in several countries, particularly in volcanic areas. Here we describe for the first time the features of endemic fluorosis in the *Herculaneum* victims of the 79 AD eruption, resulting from long-term exposure to high levels of environmental fluoride which still occur today.

**Conclusions/Significance:**

Our observations on morphological, radiological, histological and chemical skeletal and dental features of this ancient population now suggest that in this area fluorosis was already endemic in Roman times. This evidence merged with currently available epidemiologic data reveal for the Vesuvius area population a permanent fluoride health hazard, whose public health and socio-economic impact is currently underestimated. The present guidelines for fluoridated tap water might be reconsidered accordingly, particularly around Mt Vesuvius and in other fluoride hazard areas with high natural fluoride levels.

## Introduction

Fluorine is a widespread element in the earth's crust, being present in its ionic form of fluoride in a number of minerals, as well as in soil, water, plants, foods and even air [1]. During weathering and circulation of water in rocks and soils, fluorine can be leached out and dissolved in groundwater and thermal gases. Arid climate and low rainfall coupled with high evapotranspiration are the basic factors enhancing the fluoride concentration in groundwater. Potentially fluoride-rich environments are mainly linked with Precambrian basement areas and those affected by recent volcanism. [2]. Therefore, high concentrations of naturally occurring fluoride in groundwater can be found in different countries as well as locally in most parts of the world. As a result, fluoride exposure can vary markedly from one region to another [3]. In several countries, fluoride is also added to public drinking water supplies due to the benefits of low fluoride concentration intake in preventing dental caries and strengthening bones [4], [5]. Fluoride can enter public water systems from natural sources [1], [6], particularly in volcanic areas, where high rates of fluoride in drinking water are typically found due to contamination from ash deposits [5], [7], [8]. This is the case of the Somma-Vesuvius surroundings, repeatedly covered by pyroclastic products since prehistoric times [9].

Long-term intake of high doses of fluoride can have adverse effects on human health, including dental, musculoskeletal, reproductive, developmental, renal, endocrine, neurological, and genotoxic effects [5], [10], [11]. Bones and teeth are the target organs of fluoride, and tend to accumulate it with age [4], [6], [10]. Dose and duration of fluoride intake, age, sex, nutritional status and diet, climate and renal efficiency in fluoride excretion are the main factors in the development of fluorosis, an increasingly disabling disease. This disease usually affects older adults, and men more frequently than women [5], [6]. Primarily, fluoride acts as a cumulative toxin altering accretion and resorption of bone tissue and affecting the homeostasis of bone mineral metabolism, causing functional adverse effects [4]. Chronic fluorine intoxication may also induce endemic dental hypoplasia (mottled enamel), which has effects ranging from mild tooth discoloration (mottling) to severe staining, pitting and loss of enamel [7], [12], [13]. Enamel hypoplasias are caused by a wide range of environmental and genetic factors. These include malnutrition, febrile diseases, infections during pregnancy or infancy, trauma to the teeth and jaws, exposure to toxic chemicals, and a variety of hereditary disorders [14], [15]. Linear enamel hypoplasia (LEH), which may have a different aetiology than hypoplastic defects of fluorotic origin, is often utilized in palaeopathology as a systemic physiological stress indicator [16].

In bones and teeth, fluorine ion exchange takes place *via* recrystallization of hydroxyapatite into the more stable fluoroapatite, by which hydroxyl groups are replaced by fluorine ions [13]. During pregnancy, fluoride accumulates in placental tissue, acting as a partial barrier in protecting the foetus from toxic amounts of fluoride. Similarly to adults, the fluoride content of bones and teeth generally increases with advancing age of the foetus. The physiological effects of fluoride intake on the adult skeleton are the result of effects on the chemistry, gross morphology, histopathology, x-ray density, and integrity of structure of both the organic and inorganic phase of bone and teeth [13], [14].

Skeletal fluorosis is a chronic metabolic bone and joint disease caused by prolonged, excess ingestion of fluoride, mostly through water of endemic areas [17]. Increased fluoride bone content is the main indicator of fluoride poisoning [18]. Skeletal fluorosis is characterized by periosteal thickening, calcification of tendons and ligaments, and abnormal production of multiple hypertrophic bony exostoses (osteophytes) at ligamentous and muscular attachments to bone (entheses) [19]. The clinical condition exhibits bone, joint and muscle pain due to early restrictions in spine movements and at a later stage progressive ankylosis of the vertebral joints induced by ligamentous calcification [20]. Vertebrae, ribs and pelvis are more prone to osteophyte formation than long bones, even if increasing immobilization also spreads to the major joints of the chest and knees. In advanced stages the entire skeleton may be involved by crippling deformities, which can be found in the paediatric age group too [21]. Radiological and histological findings closely parallel macroscopic changes [22]. Extensive production of new bone, usually associated with bone resorption, may result in an overall increase in bone thickness and radio-opacity, besides a low degree of mineralization [23]. The outcome is a combination of osteosclerosis, osteoporosis and osteomalacia of different degrees [4], [18], [21]. An altered organic matrix, reduced mineralization and osteosclerosis are also apparent from histopathological examination [18], [24], [25]. Despite the increase in bone tissue mass but not in density, fluorotic bones are thus brittle, of poorer mechanical quality and easier to break [23], [26].

Even if skeletal fluorosis has been widely studied for more than 40 years, because some of the early clinical symptoms resemble those of osteoarthritis, the first clinical phases of skeletal fluorosis could be easily misdiagnosed [6]. In its advanced stage it becomes a crippling disability that has a major public health and socio-economic impact, affecting tens of millions of people in Africa, India and China, and being endemic in at least 25 countries across the globe [1], [6]. In studies of ancient skeletal populations, this condition has rarely been considered in differential diagnoses of palaeopathological bone lesions, mostly concerning specific single cases showing excessive ossification and joint ankylosis [27]. Fluorosis was first reported for Neolithic and Chalcolithic dental samples from Pakistan [28], [29]. Later reports for historical times [4], [30] refer to arid regions and other areas in which the disease still occurs today due to high fluoride concentrations in drinking water.

Here we present the pathological condition of a significant group of victims caught by the 79 AD Vesuvius eruption (Table 1), recently excavated on the ancient beach of *Herculaneum* [31]. All skeletons were in an extraordinary good state of preservation as a result of the unusual death and burial conditions involved: instant death caused by emplacement of hot pyroclastic surge (ca. 500°C), followed by rapid vaporization of soft tissues replaced by ash [32]. This anoxic fluoride-rich ash bed deposit was permanently saturated by groundwater [33].

**Table 1:** Sex and age at death estimates of 76 victims skeletons of the AD 79 eruption of Vesuvius.

N	Ind.	sex	age at death	N	Ind.	sex	age at death	N	Ind.	sex	age at death
1	5∶1	M	13–16	27	10∶22	M	18–23	53	12∶8	M	34–41.5
2	5∶2	F	18–22.5	28	10∶23	M	33–40	54	12∶9	F	22–28
3	5∶3	M	18–22.5	29	10∶24	F	37–45	55	12∶10	M?*	8–10
4	6∶15	?	7 iu-m	30	10∶25	M	20–23	56	12∶11	M	47–57
5	10∶1	M	36–42	31	10∶25B	?	19–24	57	12∶12	M?*	2–4
6	10∶2	M	14–16	32	10∶26	M?*	8–10.5	58	12∶13	F	33–41.5
7	10∶3	M	45–55	33	10∶27	F?*	4–6.5	59	12∶14	M*	11–13
8	10∶4	F	26–32	34	10∶28	F	33–40	60	12∶15	F	30–35.5
9	10∶5	M	18–25	35	10∶29	F	26–32	61	12∶16	M	33–41.5
10	10∶6	M	28–33.5	36	10∶30	M?*	2–3	62	12∶17	F?	5–6
11	10∶7	M	33–38	37	10∶32	M?*	8–11	63	12∶18	F?*	3–4
12	10∶8	M	11–15.5	38	10∶33	F*	11–13	64	12∶19	M	31–37
13	10∶9	M	13–16.5	39	10∶34	F?	17–22	65	12∶20	M	16–20
14	10∶10	M	33–38	40	10∶35	M	20–39	66	12∶21	F	29–35
15	10∶11A	F	28–34.5	41	10∶36	M	15–17	67	12∶22	M	18–23
16	10∶11B	M	31–37.5	42	10∶38	?	12–15	68	12∶23	M	38–46
17	10∶12	M	31–37	43	10∶39	M	12–15	69	12∶24	M?*	8–11
18	10∶13	M	31–37	44	10∶40	?	11–14	70	12∶25	M*	9–11
19	10∶14	M	34–40	45	10∶41	F?*	0.5–1.5	71	12∶26	M	28–34
20	10∶15	F	26–31	46	12∶1	M?*	3–5	72	12∶27	M	33–39
21	10∶16	F	34–38.5	47	12∶2	F	23–29	73	12∶28	F	33–39
22	10∶17	M	32–39	48	12∶3	F	25–30	74	12∶29	F?*	11–13
23	10∶18	F	35–41	49	12∶4	M	24–30	75	12∶30	F	33–37.5
24	10∶19	M	27–33	50	12∶5	M	15–18	76	12∶31	F	20–39
25	10∶20	M	40–48	51	12∶6	F?*	7–10				
26	10∶21	M	34–41	52	12∶7	M	16–18.5				

Ind. = specimen; M = male; F = female; * = probable sex in infants (0 to 12-years-old); iu-m = intra uterine months.

Due to the peculiar burial conditions of these skeletons, specific attention has been paid to discriminate pathological vs. diagenetic alteration. Bone histology, porosity, and enrichment of chemical elements are diagenetic parameters that quantify the post-mortem osteoalteration. Bones buried for long periods absorb and accumulate fluoride from soil [13], [34]. Since fluoride is taken up by bone due to interaction of bone minerals with pore water transporting fluoride, the extent of bone diagenesis is particularly related to fluctuating hydrological regimes. Furthermore, permanently waterlogged environments are anoxic, and inhibit microbial attack and related diagenetic processes [34], [35].

Being a unique cross section of the entire living population, the *Herculaneum* skeletons are particularly suited to palaeoepidemiologic investigation, which also has important implications for present-day populations. Detailed morphological, radiological, histological and chemical evaluation of the skeletal and dental features of these ancient people for the first time suggests that fluorosis was already endemic in Roman times. Our findings merged with currently available epidemiologic data strongly support the hypothesis of an enduring fluoride health hazard for the Vesuvius area population, whose public health impact is underestimated today.

## Materials and Methods

This study was approved by the Ethics Committee for Biomedical Activities of the University of Naples, Azienda Ospedaliera Universitaria “Federico II”, Naples (Protocol 154/10, 9.08.10). The Superintendency of Pompeii granted field investigation and study of the human skeletal materials unearthed in the 1997–99 excavations of the water-front chambers at *Herculaneum*.

### Morphological, x-ray and histological bone analysis

We analysed 76 human skeletons aged 0 to 52-years-old, excavated within the water-front chambers 5, 10 and 12 of the *Herculaneum* suburban area [31]. Sex and age at death, as well as the prevalence of linear enamel hypoplastic defects (LEH) and dental caries were assessed according to standard diagnostic procedures [16], [36], [37]. Enamel fluorosis was scored according to Dean's classification [38], that we simplified by adopting a four-value scoring system. The chest bones, spine, pelvis and long bones of each individual were examined for the calcification of ligaments, cartilage and tendons [39], as well as the presence of healed fractures. Hypertrophic osteosclerosis (osteophytosis) and spondyloarthritis of the spine, and osteoarthritic lesions of the appendicular skeleton were scored both individually and by single joint following standardized scoring criteria, widely applied by palaeopathologists [40], [41]. The most severe cases were also evaluated adopting digital radiography (Villa Mercury 332, Kodak Direct View CR 850, Naples, IT) and histological analysis. We examined the undecalcified and unstained bone ground sections (80–100 µm thick), obtained after embedding in LY-554 araldite resin (Vantico) and observed under transmitted ordinary and polarized light microscope. The bone histology concerning alterations of microstructure and its birefringence were also investigated in order to discriminate pathological vs. diagenetic alteration [42].

### Instrumental Neutron Activation Analysis (INAA)

The bone amount of fluorine (F), sodium (Na) and calcium (Ca) was measured in the iliac crest and/or rib bones of 27 *Herculaneum* victims aged 0 to 52-years-old of both sexes, by Instrumental Neutron Activation Analysis (INAA) [43]. The fluorine amounts (16371 ppm on average) in the infants aged 0 to 10-years-old exceeded those of 12 to 30-year-old individuals (16164 ppm on average). Therefore the former fluorine amounts were not considered for statistical elaboration. Indeed, infant bones are particularly exposed to post-mortem taphonomic as well as diagenetic processes. This is due to their porosity and lower rate of calcification [44].

### Ion-selective electrode (ISE)

The fluoride content of volcanic ash was determined at the University of Notre Dame Fluoride Dating Service, using an ion-selective electrode (ISE) according to Shurr (1989) [45].

### Statistics, skeletal lesion index

The degree of lesion involving spine and peripheral joints were evaluated by an ordinal scaling system. The degenerative changes were scored following the four-value scoring classification adopted by Jurmain [40], [41] and then standardized by means of our own lesion index.

A 0-to-3 score [p_i_] on an ordinal scale (0 = absent, 1 = moderate, 2 = severe, 3 = ankylosis) was assigned to each joint of individuals aged ≥15-years-old. The total measured score (Σ Pij = sum of scores of the j-th articulation in the i-th subject) was divided by the maximum measurable score assigned to each individual (3 • n_i_, where 3 = maximum score and n_i_ = number of joints for the i-th subject). The obtained relative and normalized skeletal lesion index (SLI) ranged from 0 to 1 (0≤SLI≤1, with 0 = absence of lesion and 1 = maximum degree of joint lesion):
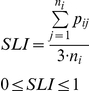



The unpreserved joints were not considered in the index calculation.

## Results

**Figure 1:**
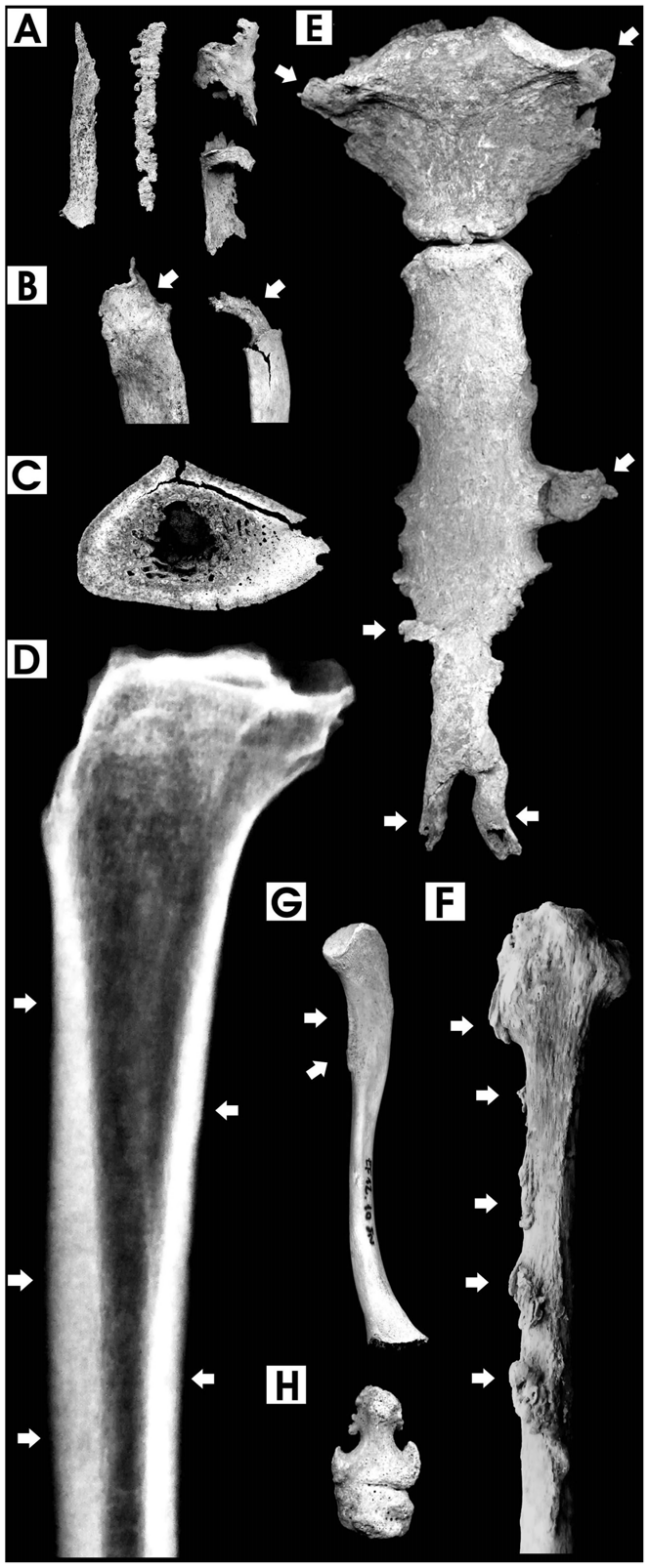
Pathological features in chest and long bones. A. Calcified ligaments and interosseous costal cartilages, 40-year-old male; B. Proximal first and sixth rib epiphyses with prominent exostoses due to interosseous cartilage calcification, 27-year-old female and 40-year-old male; C. Cross section of the mid-shaft of tibia showing extensive cortical thickening, increased bone matrix density, intracortical resorption and reduced medullary space, 40-year-old male; D. Digital x-ray image (lateral view) of the previous tibia, showing a “marble-like” appearance (arrows) symptomatic of marked osteosclerosis; E. Prominent calcification of costosternal and costoxiphoid ligament attachments (arrows) in the sternum, 40-year-old male; F. Ligamentous and interosseous membrane ossification at multiple sites (arrows) in the fibula, 40-year-old male; G. Calcification and osteophytosis at the attachment of the deltoid muscle (arrows) in the clavicle, 9-year-old male; H. Ankylosis of toe distal interphalangeal joint, 29-year-old male (bone images are in 1∶2 size).

**Figure 2:**
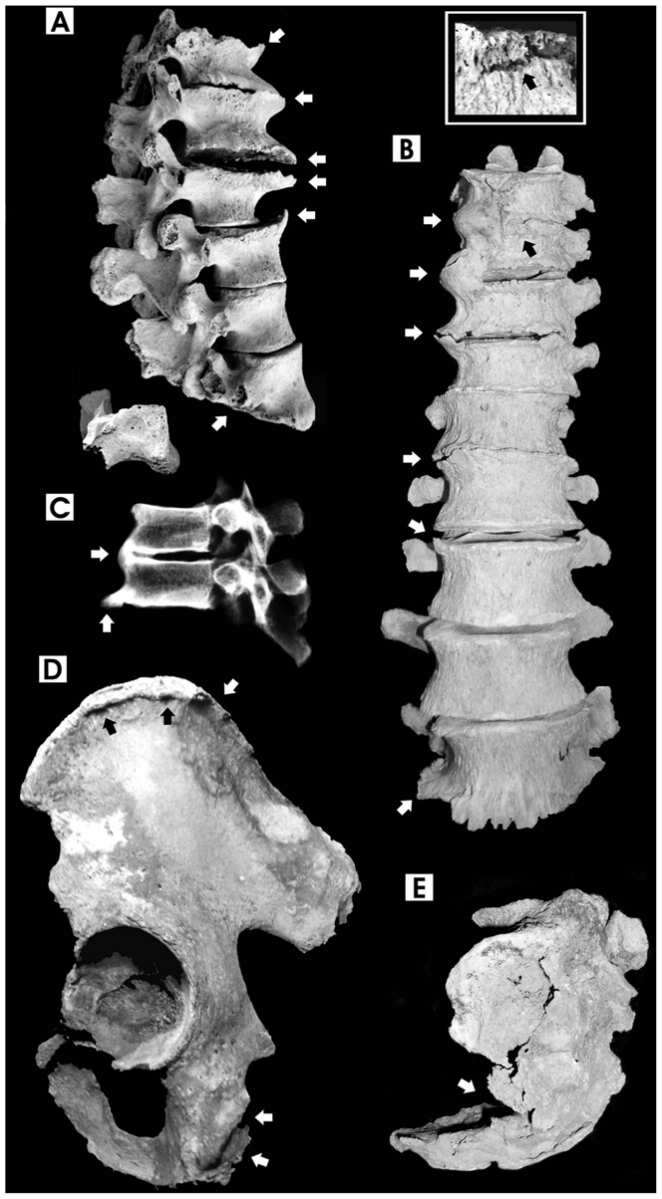
Pathological features of spine and pelvis. A. Widespread hypertrophic osteosclerosis, calcification of anterior ligaments, spondyloarthrosis and osteoporosis (arrows) of thoraco-lumbar vertebrae (T12-L5, lateral view), 44-year-old male. Notice severe flattening (osteoporosis) of the L5 vertebral body (arrow) and lumbar spondylolysis (inferior articular part split separately from the spinous process); B. Healed fracture of T10 (see enlargement in the small box), severe calcification of thoraco-lumbar anterior ligaments (T9-L5, anterior view) and ankylosis of T9-T11 vertebrae (arrows), 52-year-old male. Spondylolysis affects the L5 vertebra too; C. Digital X-ray image of T8-T9 fused vertebrae, showing diffuse osteosclerosis (lateral view), 38-year-old male; D. Ligamentous calcification and osteophytic bony spurs at the iliac crest and ischial tuberosity (sacrotuberous ligament) (arrows), 52-year-old male; E. Healed fracture of the 3^rd^ vertebra (arrow) and kyphosis of the sacrum bone (lateral view), 52-year-old male (bone images are in 1∶2 size).

At *Herculaneum*, the majority of the individuals ≥15-years-old (73.5%) show evidence of intense calcification of the ligaments, tendons, cartilage and interosseous membranes, associated with diffuse axial and appendicular osteosclerosis. Severe calcification along with proliferative bone abnormalities particularly involve costochondral and costosternal junctions (Figure 1A, E), ribs (Figure 1 B), spine anterior longitudinal ligaments (Figure 2A, B, C), iliac crest and sacrotuberous ligaments (Figure 2D) (Table S1). Gross and radiographic examination of the long bones reveal diffuse osteosclerosis in the form of massive cortical thickening, increased bone matrix density, narrowed medullary cavity and increased radio-opacity (x-ray “ebony” appearance) (Figure 1C, D). The periosteal bone also shows intracortical resorption and increased porosity, and the bones exhibit a heavy and marble-like appearance, as in incipient fossilized bones. In addition, most of the individuals (91.8%) display at least one long or flat bone affected by abnormal growth of osteophytes (Figure 1E, F) (Table S1), including several juveniles and children (Figure 1G).

**Figure 3:**
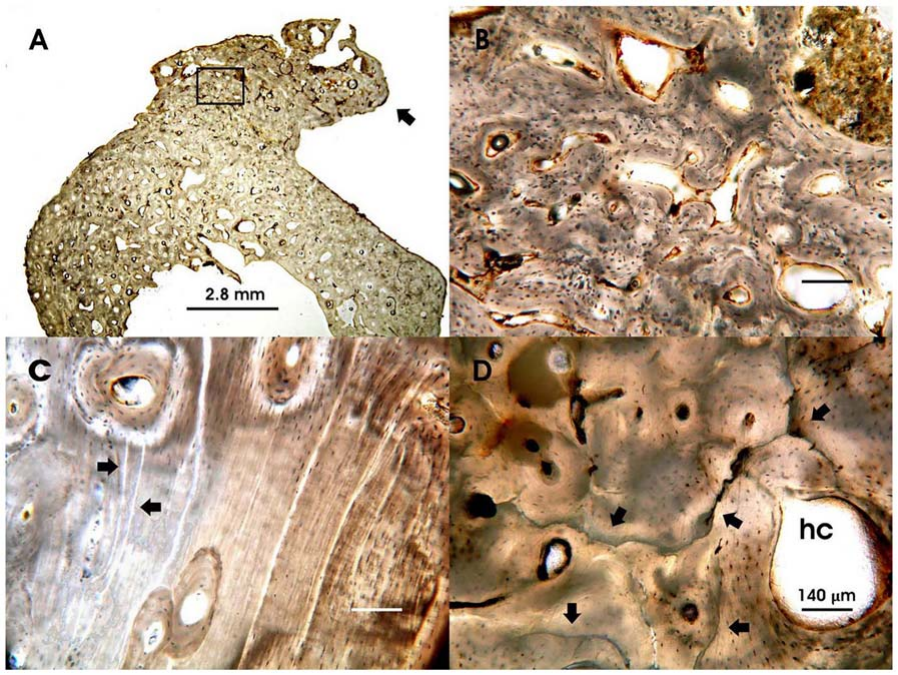
Histopathological bone features. Representative bone ground sections observed under transmitted light microscope: A. Mid shaft of fibula showing increased cortical thickness, reduced medullary cavity and a prominent exostosis (arrow) abnormally exceeding the original shape of the bone surface, 40-year-old male; B. Higher magnification of the insert of figure A, showing bone cancellization, enlarged Haversian systems and disordered lamellar architecture; C. Mid shaft of femur with widespread deficiency of the Haversian lamellar systems (arrow), extensive mottling of bone matrix and several enlarged Haversian canals, 15-year-old male. Note the presence of several linear formation defects (arrows); D. Mid shaft of tibia with osteonic texture locally poor, mottled bone matrix and an extremely enlarged Haversian canal (hc), 30-year-old female. Note the extensive and irregular cracking (arrows) induced by exposure of victims' corpses to the hot pyroclastic surge. B, C and D images are at the same magnification.

The microscopic examination of ground cross sections of the juvenile and adult long bones from both sexes show several histopathological alterations: i. increased cortical and trabecular bone thickness and massive formation of exostosis on the periosteal bone margin (Figure 3A); ii. lost or poorly formed Haversian lamellar systems (Figures 3B, C); iii. extensive mottled bone matrix and enlarged Haversian canals (Figure 3B–D). No evidence of structural diagenetic change by microorganism activity can be observed, as predictable due to the anoxic burial environment of the skeletons. A sole osteoalteration consists of widespread microcracking (Figure 3D), due to exposure of the victims' corpses to the pyroclastic surge high temperature [31], [32].

In the backbone, hypertrophic osteosclerosis and spondyloarthritis (Table S2) increase towards the lumbar spine (Figure 2A–C), with a 27.6% and 18.5% overall prevalence, and 14.5% and 5.0% of major (severe+ankylosis) joint lesions, respectively. Spine ankylosis, mostly due to anterior ligament calcification (Figure 2B, C), mainly involves thoraco-lumbar (3.1%) and sacroiliac (4.6%) joints, affecting males and females equally (18.2% vs. 17.6% of the individuals, z = 0.0523, P>0.05) (Table S1). In these severe cases x-rays appear homogeneously dense, the vertebral body contours are largely uneven or fused, and bones have a chalky white appearance (Figure 2C).

Furthermore, a 47.2% overall occurrence of osteoarthritic-like lesions (14.9%, severe+ankylosis major lesions) involving the joints of the appendicular skeleton appears particularly severe considering the mean age of 30.2 years (individuals ≥15-years-old). The coxofemoral, knee, sacroiliac, elbow, and sternoclavicular joints and pedal phalanges are the most noticeably affected anatomical districts (Table 2). In general, ankylosis affects at least one anatomical site in 39.2% of the individuals, involving mainly the spine (Figure 2B, C), the distal interphalangeal joints of toes (Figure 1H) and manubriosternal joints (Table S1). In addition, nearly one individual out of three (32.1%) shows one or more pathologic fractures involving mostly the spine (Figure 2B) or os coxa (Figure 2E), as well as long bones, while osteomalacia affects 8.2% of the individuals. Evaluating the cases of spondylolysis (L5 vertebrae, 8.9% vs. 3–7% of the general population) as stress fractures (Figure 2A) [46], [47], the susceptibility to bone fractures is particularly high at *Herculaneum* (35.7%) (Table 3).

**Table 2:** Occurrence of osteoarthritic lesions in 737 joints of the appendicular skeleton of individuals aged ≥15-years-old.

	Sternoclavicular			Shoulder			Elbow			Wrist			Hand		
	L	R	%	L	R	%	L	R	%	L	R	%	L	R	%
Absent	20	20	*54.1*	11	11	*29.7*	14	17	*37.4*	15	19	*48.6*	10	12	*81.5*
Traces	1	3	*5.4*	4	4	*10.8*	9	2	*13.3*	10	5	*21.4*	3	0	*11.1*
A+T %	*58.3*	*64.7*	*59.5*	*36.6*	*45.5*	*40.5*	*52.3*	*48.7*	*50.6*	*69.4*	*70.6*	*70*	*100.0*	85.7	*92.6*
Moderate	10	12	*29.7*	24	13	*50*	18	14	*38.6*	10	9	*27.1*	0	0	*0*
Severe	5	3	*10.8*	2	5	*9.5*	3	6	*10.8*	1	1	*2.9*	0	2	*7.4*
Ankilosys	0	0	*0*	0	0	*0*	0	0	*0*	0	0	*0*	0	0	*0*
M+S+A %	*41.7*	*39.5*	*40.5*	*63.4*	*54.5*	*59.5*	*47.7*	*51.3*	*49.4*	*30.6*	*29.4*	*30.0*	*0.0*	*14.3*	*7.4*
S+A %	*13.9*	*7.9*	*10.8*	*4.9*	*15.2*	*9.5*	*6.8*	*15.4*	*10.8*	*3.1*	*2.9*	*2.9*	*0.0*	*14.3*	*7.4*
total %			*100.0*			*100.0*			*100.0*			*100.0*			*100.0*
N	36	38	74	41	33	74	44	39	83	36	34	70	13	14	27

	Sacroiliac			Coxofemoral			Knee			Ankle			Foot		
	L	R	%	L	R	%	L	R	%	L	R	%	L	R	%
Absent	18	17	*41.2*	13	15	*31.1*	10	12	*26.2*	17	17	*38.6*	13	15	*45.2*
Traces	2	6	*9.4*	3	6	*10*	10	6	*19*	5	8	*14.8*	4	2	*9.7*
A+T %	*47.6*	*53.5*	*50.6*	*34.8*	*47.7*	*41.1*	*46.5*	*43.9*	*45.2*	*50*	*56.8*	*53.4*	*54.8*	*54.8*	*54.8*
Moderate	15	11	*30.6*	20	13	*36.7*	16	14	*35.7*	19	15	*38.6*	3	2	*8.1*
Severe	5	8	*15.3*	10	10	*22.2*	7	9	*19.1*	3	4	*8*	6	6	*19.3*
Ankilosys	2	1	*3.5*	0	0	*0*	0	0	*0*	0	0	*0*	5	6	*17.7*
M+S+A %	*53.4*	*46.5*	*49.4*	*65.2*	*52.3*	*58.9*	*53.5*	*56.1*	*54.8*	*50.0*	*43.2*	*46.6*	*45.2*	*45.2*	*45.2*
S+A %	*16.7*	*20.9*	*18.8*	*21.7*	*22.7*	*22.2*	*16.3*	*21.9*	*19.1*	*6.8*	*9.1*	*8*	*35.5*	*38.7*	*37.1*
total %			*100.0*			*100.0*			*100.0*			*100.0*			*100.0*
N	42	43	85	46	44	90	43	41	84	44	44	88	31	31	62

	Total		
	L	R	%
Absent	141	155	*40.2*
Traces	51.0	42.0	*12.6*
A+T %	*51.1*	*54.6*	*52.8*
Moderate	135	103	*32.3*
Severe	42.0	53.0	*13.0*
Ankilosys	7.0	7.0	*1.9*
M+S+A %	*48.9*	*45.4*	*47.2*
S+A %	*13.0*	*16.9*	*14.9*
total %			*100.0*
N	376	361	737

N = number of articulations; L = number of left articulations; R = number of right articulations.

**Table 3:** Occurrence of healed bone fractures in 56 individuals aged ≥15-years-old.

Individual	sex	age at death	bone
5∶3	M	18–22.5	clavicle (right)
10∶22	M	18–23	hand phalanx (right)
12∶9	F	22–28	foot phalanx (right)
10∶15	F	26–31	rib 11 (left)
10∶35	M	20–39	fibula (left)
12∶SP1	M?	20–39	tibia (right)
12∶26	M	28–34	spondylolysis L5 vertebra
10∶11A	F	28–34.5	ox coxae - head of femur (right)
12∶19	M	31–37	foot phalanx (right)
12∶30	F	33–37.5	T11 vertebra
10∶16	F	34–38.5	clavicle (right)
10∶23	M	33–40	spondylolysis L5 vertebra
12∶16	M	33–41.5	clavicle (right) - foot phalanx (left)
12∶13	F	33–41.5	rib 7 (right)
12∶8	M	34–41.5	foot phalanx (right)
10∶1	M	36–42	clavicle (left)
10∶24	F	37–45	T8 vertebra - foot phalanx (right)
12∶23	M	38–46	T12 vertebra - spondylolysis L5 vertebra
10∶3	M	45–55	spondylolysis L5 vertebra - foot phalanx (left) - radius (left)
12∶11	M	47–57	T10 vertebra - spondylolysis L5 vertebra - sacrum - rib 6 (left)

**Figure 4:**
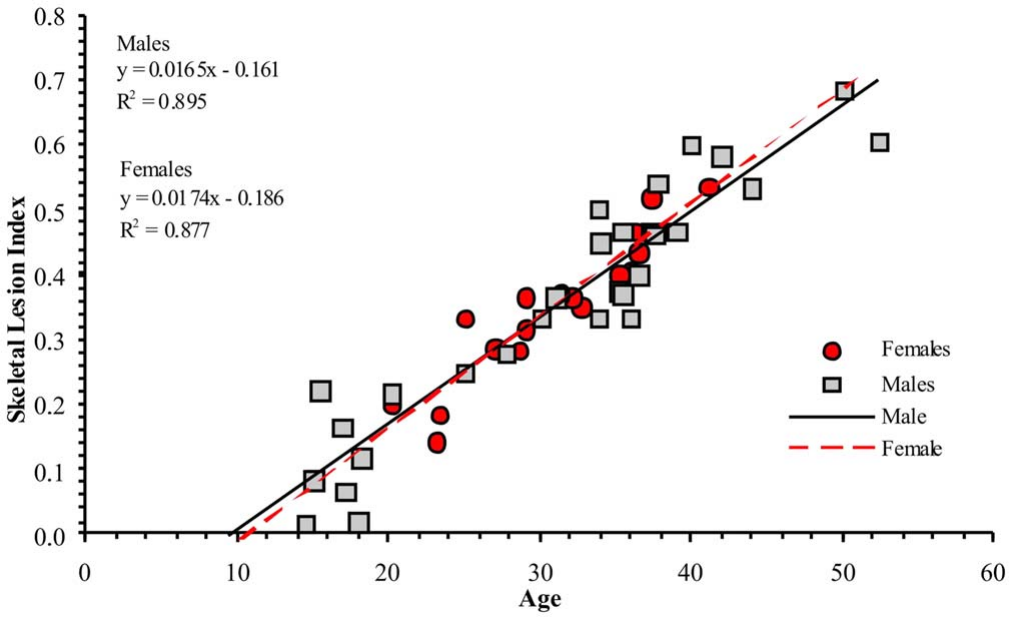
Skeletal lesion index related to age by gender. The linear regressions obtained by comparing skeletal lesion index (SLI) vs. age, separately for males and females, shows that nearly 90% of SLI variability is age related (males: R^2^ = 0.895, P<0.0001; females: R^2^ = 0.877, P<0.0001). However, males aged ≤30-years-old are in average more affected than females, while females over the thirties are more frequently involved (test for no equality of regression coefficients, t = 7, P<0.0001).

The degree of individual osteoarthritic-like lesions involving the post-cranial skeleton was also assessed by means of a skeletal lesion index (SLI). The SLI index was adopted to better evaluate and compare expression and variability of pathological lesions as to anatomical location, age and gender. This index, calculated by considering both appendicular skeleton and spine joints of the individuals aged ≥15-years-old (Table 4), was regressed by individual age, separately for males and females (Figure 4). In both sexes the linear regression shows that nearly 90% of the SLI index variability is age-related (males: R^2^ = 0.895, P<0.0001; females: R^2^ = 0.877, P<0.0001), but the slope differs significantly between males and females (test for no equality of regression coefficients, t = 7, P<0.0001). Males ≤30-years-old are on average more affected than females, while females over 30 seem more prone to be involved. SLI indexes calculated on 10 post-cranial large joints, show that the spine is by far the most affected (0.616) (z = 2.15, P<0.05), compared with knee (0.356), shoulder (0.317), coxofemoral (0.306), sacroiliac (0.284) and other joints.

**Table 4:** Skeletal Lesion Index calculated on post-cranial joints in 48 individuals aged ≥15-years-old.

N	Ind.	Age	Sex	St-Cl	Shou	Elbow	Wrist	Sa-Il	Co-Fe	Knee	Ankle	Foot	Spine	I	I_max_	I_norm_
1	12∶2	23.2	F		1	1		0	0		0	0	1	3	21	0.143
2	12∶3	23.4	F		0	0	0	1	1	2	0	0	1	5	27	0.185
3	12∶4	27.0	F	1				0.5	1	0	0.5	1	2	6	21	0.286
4	12∶5	15.2	M						0	0	0		1	1	12	0.083
5	12∶7	17.0	M			0		0	0	0	0	3		3	18	0.167
6	12∶8	37.8	M		1	1	0		2	3	1	3	2	13	24	0.542
7	12∶9	25.0	F	1	1	1	0	0	0	1	1	3	2	10	30	0.333
8	12∶11	52.3	M	2	0.5	3		2	2		2	0	3	14.5	24	0.604
9	12∶13	37.3	F		1	1	1	2	2	2	2	0	3	14	27	0.519
10	12∶15	32.7	F	1	1	1	0.5	0	1	1	1	2	2	10.5	30	0.350
11	12∶16	37.2	M	2	1	1	1	0	1	1	1	3	3	14	30	0.467
12	12∶19	33.9	M	2	1	1	1	0	0	1	1	1	2	10	30	0.333
13	12∶20	18.0	M	0	0	0	0.5	0	0		0	0	0	0.5	27	0.019
14	12∶21	32.1	F	0	0	1	0	0	2	2	1	3	2	11	30	0.367
15	12∶22	17.2	M	0	0	0	0	0	0	0	1	0	1	2	30	0.067
16	12∶23	42.0	M	2		1		1	1	2	1	3	3	14	24	0.583
17	12∶26	31.0	M	2	1	0	0	0	2	1	0	2	3	11	30	0.367
18	12∶27	36.0	M	2	1	1	1	0	0	1	1		2	9	27	0.333
19	12∶28	36.0	F	2		1	1	0	1	1	0	3	2	11	27	0.407
20	12∶30	35.2	F	2	2	1	1	1	1	0	1	1	2	12	30	0.400
21	5∶01	14.5	M	0	0	0	0	0.5	0	0	0	0	0	0.5	30	0.017
22	5∶02	20.2	F	1	1	0	0	0	0	1	2	0	1	6	30	0.200
23	5∶03	20.2	M	1	1	0	0.5	0.5	1	0.5	0	0	2	6.5	30	0.217
24	10∶1	39.0	M	1	2	2	1	1	2	1	1	0	3	14	30	0.467
25	10∶2	15.0	M	0	0	0	0	0	0	0.5	1	0	1	2.5	30	0.083
26	10∶3	50.0	M	3	2	0.5	1	3	2	1	2	3	3	20.5	30	0.683
27	10∶4	29.0	F	0.5	0.5	1	0.5	1	1	1	1	1	2	9.5	30	0.317
28	10∶5	18.2	M	1	0	0	0	0	0	0	0.5	1	1	3.5	30	0.117
29	10∶6	27.8	M	1	1	0.5		0	1	0	1	2	1	7.5	27	0.278
30	10∶7	35.5	M	1	1	1	1	2	1	1	1	3	2	14	30	0.467
31	10∶10	35.1	M	1	2	0		2	0	1	1		2	9	24	0.375
32	10∶11	31.3	F	1	1	1	0.5	1	2	1	0.5		2	10	27	0.370
33	10∶12	34.0	M	1	1	1	0.5	2	1	1	1	3	2	13.5	30	0.450
34	10∶13	33.9	M	1	1	2	1	2	1	2	1	2	2	15	30	0.500
35	10∶14	40.0	M	2	1	2	2	1	2	2	1	2	3	18	30	0.600
36	10∶15	28.6	F	2	1	1	0	1	1	1	0	0.5	1	8.5	30	0.283
37	10∶16	36.3	F	2	1	1	1	2	1	1	1	2	2	14	30	0.467
38	10∶17	35.5	M	2	2	0	1	1	1	1	1		1	10	27	0.370
39	10∶18	38.0	F	2	0	1	1	1	0	2	2	3	2	14	30	0.467
40	10∶19	30.0	M	2	2	1	0	1	0	1	1	1	1	10	30	0.333
41	10∶20	44.0	M	2	2	2	1	1	1	2	1	2	2	16	30	0.533
42	10∶21	37.5	M	2	1	2	1		2	1	1	0.5	2	12.5	27	0.463
43	10∶22	15.5	M	0	0	1	0	1	1	1	0		2	6	27	0.222
44	10∶23	36.5	M	2	1	1	1	1	1	1	1	1	2	12	30	0.400
45	10∶24	41.1	F		1	1	0.5	2	2	2	2	2	2	14.5	27	0.537
46	10∶25	25	M						0	2	0	1		3	12	0.250
47	10∶28	36.5	F	1	2	2	0	2	2	1	1	0	2	13	30	0.433
48	10∶29	29	F	1	1	1	1	1	1	1	1	1	2	11	30	0.367

Ind. = specimen; St-Cl = sternoclavicular; Shou = shoulder; Sa-Il = sacroiliac; Co-Fe = coxofemoral; I = total measured score; I_max_ = maximum measurable score; I_norm_ = skeletal lesion index.

**Figure 5:**
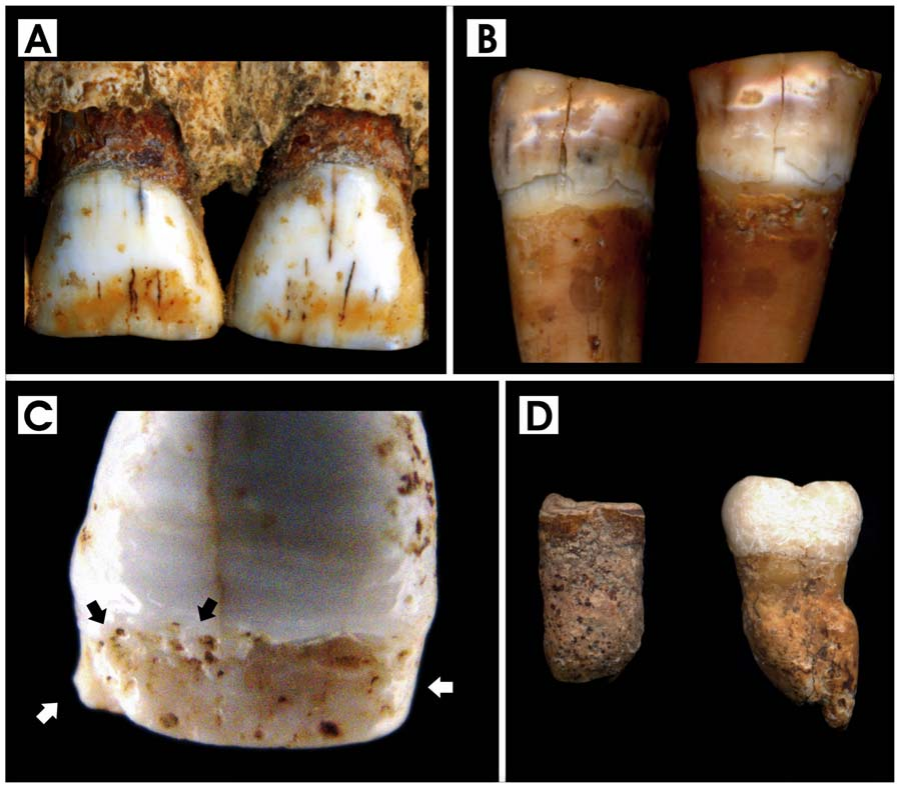
Dental pathological features. A. Yellow-brown stains, upper central incisors, male 36-years-old; B. Mottling and brown staining, lower premolars, male 50-years-old; C. Discrete and confluent pitting (black arrows), upper left central incisor, male 20-years-old. Note the severe enamel hypomineralisation in the form of corroded-like appearance (white arrows); D. Hypercementosis of roots in lower molars, males 26-years-old (left) and 30-years-old (right).

Analysis of permanent dentition reveals 96.1% of the individuals (47.3% of teeth) affected by linear hypoplastic defects (LEH) (Table 5). In addition, mottling, pitting and/or staining of the enamel (Figure 5A, B, C) was found in 53.1% of the sample (54.9% of teeth), with moderate to severe enamel alterations involving 34.4% of the individuals (27.6% of teeth) (Table 6). In the cases of marked hypomineralization (25.0%, 17.8% of teeth), a corroded-like appearance and alterations of the tooth form are evident (Figure 5C). Mottled enamel, associated with chronic dental fluorosis since prehistoric times [28], [29], may also affect well nourished people. A healthy diet for the *Herculanenses* is testified by historical and archaeological evidence [48], as well as from trace-element analyses of a previously excavated group of victims (Herc2) [33], [49]. Carious lesions, collected as an additional test of dental pathological status, are found in 20% of permanent teeth and 78.6% of individuals (Table 5). A few cases of root hypercementosis have also been detected (Figure 5D).

**Table 5:** Occurrence of caries and linear enamel hypoplasia (LEH) in permanent teeth.

	Caries		LEH	
Tooth	N	%	N	%
I1	182	2.2	136	64.7
12	195	5.1	146	68.5
C	179	3.9	134	83.6
P3	176	10.2	132	37.9
P4	168	27.4	129	31.8
M1	178	33.7	126	27.8
M2	168	44.0	134	28.4
M3	112	47.3	71	18.3
Total	1358	20.0	1008	47.3

individuals affected by caries 78.6% (N = 56).

individuals affected by linear enamel hypoplasia 96.1 (N = 51).

**Table 6:** Evaluation of hypoplastic mottling in enamel of permanent teeth.

Individual	M-WA	BS	P	CP	CLA	minimum ind. score	maximum ind. score	average ind. score	general evaluation
5.1	2.0	1.0	2.0	1.0	2.0	1.0	2.0	1.4	moderate
5.3	1.7	1.2	1.6	1.4	1.3	1.2	1.7	1.4	mild
10.2	0.7	0.8	0.8	0.6	0.6	0	0.8	0.7	normal
10.3	1.8	2.6	1.9	1.3	1.0	1.0	2.6	1.9	severe
10.5	0	0.3	0.9	0.1	0	0	0.9	0.3	normal
10.6	0.3	0.4	1.0	0.4	0.2	0.2	1.0	0.5	normal
10.7	1.0	0.3	1.1	0.7	0.7	0.3	1.1	0.8	mild
10.12	0.7	0.3	0.8	0.1	0.2	0.1	0.8	0.4	normal
10.13	0.4	0.4	0.7	0.4	0.3	0.3	0.7	0.4	normal
10.14	1.0	2.0	1.0	1.0	1.0	1.0	2.0	1.2	moderate
10.15	0.9	0	1.1	0.5	0.4	0	1.1	0.6	mild
10.17	1.4	1.9	1.3	0.5	0.7	0.7	1.9	1.2	moderate
10.19	1.3	2.3	2.0	1.7	1.7	1.3	2.3	1.8	severe
10.20	1.6	1.4	1.4	1.1	0.8	0.8	1.6	1.3	mild
10.22	1.0	0.8	2.0	1.6	1.6	0.8	2.0	1.4	moderate
10.23	1.6	1.6	1.6	1.3	1.3	1.3	1.6	1.5	moderate
10.24	0.4	0.4	0.1	0	0	0	0.4	0.2	normal
10.26	0.5	0	0.3	0	0	0	0.5	0.2	normal
10.28	2.0	0	1.5	0.5	0	0	2.0	0.8	moderate
10.SPD1	3.0	3.0	3.0	1.0	/	1.0	3.0	2.0	severe
10.SPD2	1.0	0	1.0	0	0	0	1.0	0.4	normal
10.SPD3	1.0	/	0	0	0	0	1.0	0.3	normal
12.5	0.8	0.3	0.6	0.3	0.3	0.3	0.8	0.5	normal
12.15	1.2	0.5	1.6	1.5	1.6	0.5	1.6	1.3	mild
12.16	0.6	0.3	0.8	0.2	0.2	0.2	0.8	0.4	normal
12.19	0.6	0	0.9	0.5	0.5	0	0.9	0.5	normal
12.22	1.8	1.5	2.3	2.1	2.3	1.5	2.3	2.0	severe
12.23	2.5	2.8	1.8	1.3	0.9	0.9	2.8	1.9	severe
12.26	0	0	0	0	0	0	0	0	normal
12.27	0.7	0.4	0.7	0.3	0.1	0.1	0.7	0.4	normal
12.28	1.6	1.2	1.2	0.6	0.4	0.4	1.6	1.0	mild
12.30	0	0	0	0	0	0	0	0	normal

M-WA = milky-white appearance; BS = yellow-brown stains; P = pitting; CP = confluent pitting; CLA = corroded-like appearance;

0 = normal (translucent and smooth enamel, glossy appearance).

1 = mild (scattered small, opaque, milky-white patches; faint brown stains are sometimes apparent);

2 = moderate (diffuse white opaque areas, minute pitting; brown stain is frequent; surfaces subject to attrition show marked wear).

3 = severe (pits deeper and confluent, widespread stains; the tooth show a corroded-like appearance).

/ = indefinable.

In order to further assess the pathological conditions of the population of ancient *Herculaneum*, we measured by Instrumental Neutron Activation Analysis (INAA) the fluorine (^19^F) bone concentrations in a large representative group of victims selected by anatomical district (iliac crest or rib), age and state of preservation (Table 7). Volcanic ash samples from the three investigated chambers were also tested by ion-selective electrode (ISE). Adjusted values of μ fluoride (160±33, 190±18, 200±6, ppm) do not diverge statistically, being included within the normal score limits (±1.96).

**Table 7:** Human bone samples tested for determination of fluorine concentration.

Ind.	Average age	Skeletal element	Na-corrected μ F (ppm)			corrected F values	± E.S.V.I. 95%
11∶15	0 (7 iu-m)	Rib	18400	±	1275	/	/
10∶41	0.5–1.5	Ilium	16600	±	540	/	/
12∶12	2.5–3.5	Ilium	16900	±	450	/	/
12∶18	3–4	Ilium	15200	±	793	/	/
12∶17	5–6	Ilium	16500	±	476	/	/
12∶6	8–9	Ilium	16100	±	1803	/	/
12∶25	9–11	Ilium	14900	±	1340	/	/
12∶14	11–13	Ilium	14000	±	1267	2042	3392
5∶1	13–16	Ilium	14400	±	372	2442	3328
12∶20	16–20	Ilium	16000	±	624	4042	3249
12∶9	23–28	Rib	16300	±	467	4342	3138
12∶4	24–30	Rib	17200	±	381	5242	3118
10∶29	26–32	Rib	16350	±	166	4392	3103
10∶19	27–33	Rib	18900	±	435	6942	3098
12∶26	28–34	Ilium	17300	±	597	5342	3094
10∶13	31–37	Ilium	19000	±	495	7042	3090
10∶12	31–37	Ilium	19250	±	311	7292	3090
12∶28	32–39	Ilium	19300	±	271	7342	3095
12∶27	32–39	Ilium	20800	±	197	8842	3095
10∶14	34–40	Ilium	18200	±	398	6242	3099
10∶21	34.5–40.5	Ilium	19200	±	213	7242	3102
10∶18	35–41	Rib	19850	±	221	7892	3105
10∶1	36–42	Ilium	22100	±	363	10142	3113
12∶23	38–46	Ilium	19900	±	620	7942	3142
10∶20	40–48	Ilium	23050	±	508	11092	3169
10∶3	45–55	Rib	23300	±	95	11342	3279
12∶11	47–57	Ilium	18200	±	196	6242	3325

Ind. = specimen; iu-m = intra-uterine months; E.S.V.I. = interval for expected single value.

**Figure 6:**
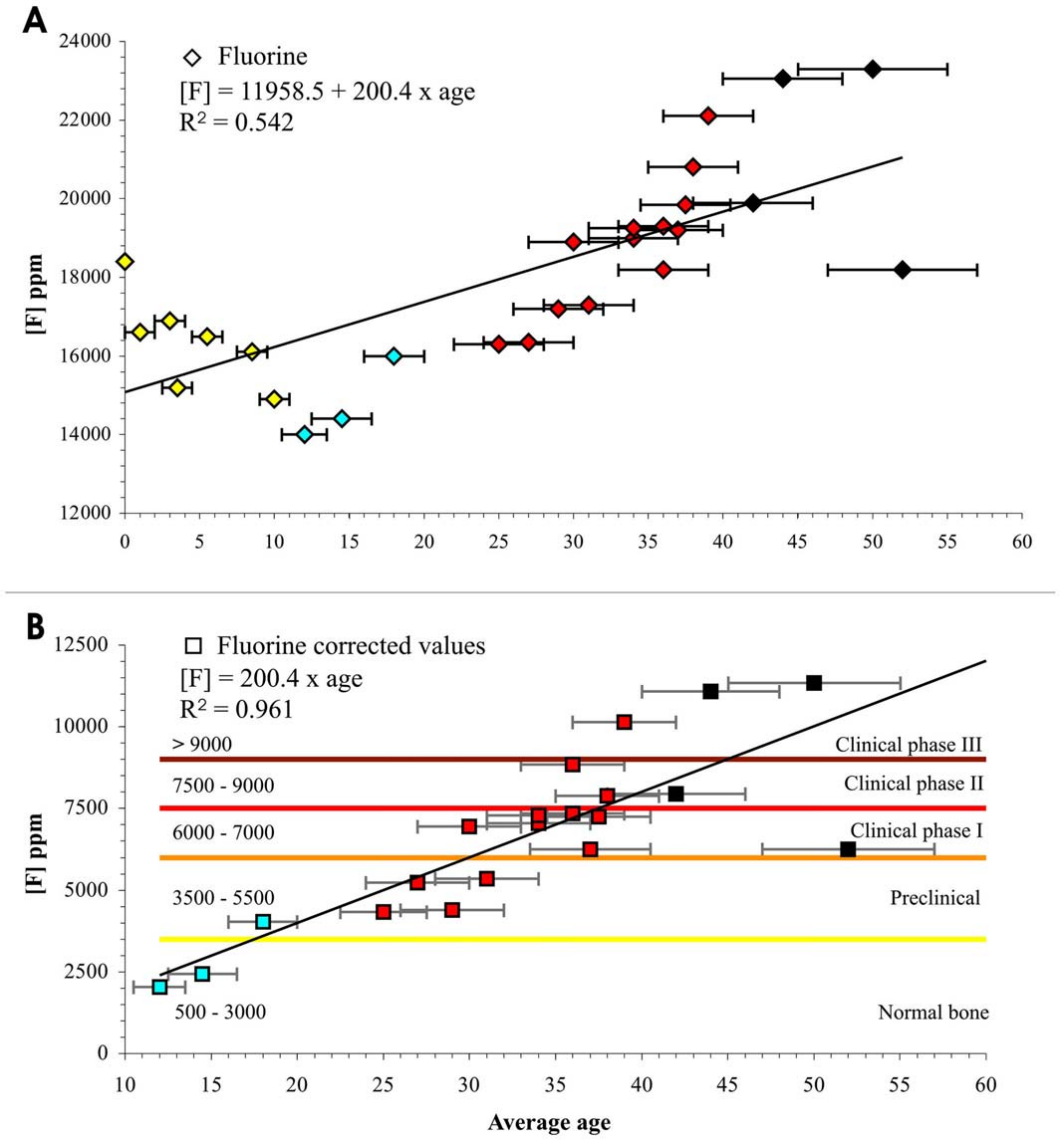
Fluorine (^19^F) bone concentration (ppm) as a function of age. The linear regression resulting from (A) the fluorine mean amount of 18400 to 23300 ppm measured by INAA (intercept = 11958.5±1120, P<0.001) is compared with an equivalent regression (B) obtained considering a 0 fluorine concentration at age 0 (slope = 200.4±9, P<0.001). The last model, representing the physiological rate of individual intake per year cleansed of the fraction of fluorine contamination by soil ash deposit, shows an evident age-dependent increase of fluorine (R^2^ = 0.961). Children aged ≤10-years-old were not included in this model, due to the high diagenetic amount of fluorine released by the ash deposit. The resulting corrected mean values of 2042 to 11342 ppm show a minority of individuals matching the normal-physiological (<3500 ppm) and preclinical (<5500 ppm) ranges of fluorine bone concentration, while the majority belongs to all the three clinical phases of skeletal fluorosis, with several mature (≥40-years-old) individuals in the crippling phase III.

The average bone fluorine concentrations varying between 14000 and 23300 ppm are a function of age as shown by the equation 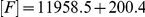 • age. The intercept (b_0_ = 11958.5±1120; P<0.001) represents the mean amount of fluorine at age 0 (Figure 6A). These values clearly exceed the range of normal-physiological fluorine bone content [50], [51] as well as the maximum expected pathological levels [6], [7]. This finding is consistent with the particular burial conditions of the skeletons. The ash deposit was permanently waterlogged [33], and the bones were therefore enriched with fluoride leaching from the groundwater. In this area the maximum concentration of present-day groundwater fluorine is 3.6 mg/L. Thus, in order to discriminate the *post-mortem* from *intra-vitam* fluoride bone enrichment, we calculated a new regression equation. We assumed a 0 fluorine concentration at age 0 (Figure 6B), considering that new-born bone usually contains nearly 50 ppm fluoride [13]. The slope (b_1_ = 200.4±9; P<0.001) from the resulting straight-line equation [F] = 200.4 • age represents the rate of physiological intake per individual per year. This model shows that 99% of fluorine concentration variability is age-related (R^2^ = 0.961).

Taking into account this last correlation and that the skeletal lesion index is fluorine concentration-related (R^2^ = 0.923), we obtained a new equation combining
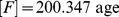
(1)and
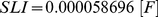
(2)thus yielding
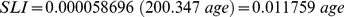
(3)This equation, applied to the available data, shows the correlation (R^2^ = 0.81) of the observed data with those expected. This indicates that the SLI index suitably describes the degree of joint lesions shown by the *Herculaneum* people as a result of the fluorine accumulating in their bones.

## Discussion

The overall evidence at *Herculaneum* clearly shows the shape of an endemic system-disease, affecting both adults and subadults, characterized by diffuse osteosclerosis and enthesopathy. Although these conditions may be associated to other bone disorders, the concomitant aberrant growth of new bone, ligamentous calcification and osteosclerosis, along with osteoarthritic-like lesions and ankylosis of spine and appendicular joints, strongly suggest skeletal fluorosis [4], [5], [52]. In addition, histopathological bone features like increased cortical thickness, abnormal lamellar texture, disordered lamellar orientation, extensive mottling of the bone matrix, enlarged and poorly formed Haversian systems, are highly characteristic of skeletal fluorosis [23], [24], [25]. The high occurrence of bone fractures and a few cases of osteomalacia are also typical of fluoro-osteoporotic bones, likely resulting from calcium deficiency [23], [52] and other mineral abnormalities induced by fluoride [24]. Furthermore, a major result is the significant correlation between the number of bone fractures (1 to 4) per individual and age (Spearman's rank correlation, r = 0.647, P<0.005) (Table 3). The widespread calcification of the sacrotuberous ligament and a few cases of tooth hypercementosis also confirm the diagnosis of skeletal fluorosis [4], [52].

A distinctive result is the high levels of fluoride found in the victims' bones, whose corrected average values ranging from 2042 to 11342 ppm (mean value ± SE: 6672±570 ppm) clearly indicate fluoride poisoning. The fluorine concentration as a function of age (Figure 6B) shows a minority of individuals with normal-physiological (<3500 ppm) and preclinical (<5500 ppm) fluorine levels, while the majority belong to all three clinical phases of skeletal fluorosis. Higher values (>9000 ppm) observed in mature adults (≥40-years-old) can be ascribable to the crippling phase III [6], as seen at present in endemic regions [53].

The regression line describing the annual variation rate of fluoride (ppm/years) shows a significant increase in fluoride concentration with age, and a correlation with the degree of pathological involvement of the spine and appendicular joints, as assessed by SLI index evaluation. This correlation of bone fluoride concentration with both duration of exposure and extent of bone lesions has been demonstrated in present-day patients affected by skeletal fluorosis, the severity of which was found to be related to the amount of fluoride incorporated into bone. Usually, fluorine can range from ca 500 to ca 3000 ppm in unaffected people, exposed to optimal fluoride intakes ≤1 mg/L [50], [51]. Instead, extreme high values of ca 10000 or 12000 ppm are typically associated with crippling fluorosis due to exposure to fluoride intakes ≥4 mg/L [5], [7]. At *Herculaneum*, the difference by gender in skeletal lesion occurrence in relation to age would merit further investigation, given the higher occurrence of skeletal fluorosis in females >30 contrasting with the epidemiological evidence [4], [6], [13], [18].

The widespread occurrence of osteoarthritis, osteophytosis, enthesopathy and fractures is particularly high in comparison with other Roman and pre-Roman communities [54], [55], even with those of low social status [56]. Palaeopathologic investigation of the Herc2 specimens confirms the high occurrence of degenerative joint disease, long bone osteosclerosis, enthesopathy and trauma [33], [46], [49], [57]. In contrast, an analogous pattern of skeletal changes, but with a lower occurrence and involving older individuals has been reported in ancient Arabic people, also affected by dental fluorosis. These arid regions are characterized by medium-high concentrations (0.5 to 3.0 mg/L) of fluoride in water [4], [30]. Dental fluorosis has first been reported for early Neolithic at Mehrgarh, Pakistan. Groundwater samples from this arid area show 1.9–2.0 mg/L of natural fluoride [28], [29]. At *Herculaneum*, the concentration of fluoride in groundwater was found to be even higher (3.6 mg/L). But despite this fact, dental fluorosis appears less severe when compared with that from Mehrgarh, most likely as a result of the lower need of hydration in the more temperate Mediterranean climate. The prevalence of mottled enamel alterations at *Herculaneum* parallels the pathological condition exhibited by the victims' skeletons. Thus, the occurrence of endemic dental hypoplasia appears correlated to high fluoride intake during life, inferred from the high amount of fluoride that we determined in the bones.

In ancient *Herculaneum* enamel mottling is associated with high levels of linear enamel hypoplasia (LEH), which occurs commonly in most ancient populations [16], [55]. Also other Roman communities, including the Herc2 sample, show constantly high rates of LEH, independently of socio-economic status [33], [54], [55], [56], [58], [59]. Considering the possible benefits of fluoride intake in preventing dental decay, caries occurrence appears unusually high if compared with other Roman Imperial age communities [49], [58], [59]. Consistently with recent epidemiologic studies [7], [60], the estimated high fluoride intake and the resulting hypomineralization of tooth enamel appear to have increased the risk of caries for the *Herculaneum* residents.

This palaeoepidemiologic scenario has likely remained unchanged for the Vesuvius area population till today. Currently, the maximum fluorine concentration of the water-bearing stratum is close to the current Maximum Contaminant Level (MCL) of 4 mg/L of drinking water, and within the range of concentrations able to induce crippling skeletal fluorosis [11], [61]. We calculated a fluoride intake of 10.8–18.0 mg/day per person at the time of the AD 79 eruption, which is equivalent to the total intake of 10–20 mg/day over a 10–20 year period, commonly associated with crippling skeletal involvement [9], [61], [62] and able to increase the risk of bone fractures [6], [52].

At present, in volcanic and other areas where groundwater is contaminated with fluoride of natural origin, communities with normal nutritional intake exposed to fluoride water concentrations of ca 4 mg/L and daily total intake of ca 14.0 mg/day show prevalence of skeletal and dental fluorosis equivalent to those observed for the *Herculaneum* residents [52], [53], [63], [64]. Notably, lower concentrations of ca. 2.0 mg/L of fluoride present in the Bolan and Nari rivers in Baluchistan, Pakistan, have been found associated with severe cases of dental fluorosis in modern and ancient people of this arid region [28], [29]. Furthermore, extensive research from India [6] provides evidence that endemic skeletal fluorosis can occur at water-borne fluoride concentrations of 2–3 mg/L or even as low as 1.1–1.5 mg/L, with crippling deformities appearing at 2.8 mg/L, given the presence of predisposing factors (geology, soil, climate, groundwater chemistry).

The overall epidemiologic scenario that we detected for the ancient inhabitants of *Herculaneum* unequivocally points to endemic skeletal and dental fluorosis induced by environmental fluoride poisoning, still occurring today. A clinical-epidemiological investigation in schoolchildren from the Vesuvian towns [65], where the maximum fluoride content in tap water was 2.8 mg/L according to local guidelines [66], found 80% prevalence of dental fluorosis - commonly considered a biomarker for fluoride exposure [13] - and related clinical features of epidemic significance. The children, aged 7 to 11 years old, were affected by stomachache, blood vessel dilatation, hair loss, articular pains and dermopathies of different degrees as well as some cases of borderline hyperthyroidism. The fluoride content in all blood samples greatly exceeded both the normal and pathological levels, as well as the World Health Organization (WHO) recommended maximum levels [3].

A recent report by the National Academy's National Research Council (NRC) [7] concluded that the Maximum Contaminant Level (MCL) of 4 mg/L of fluoride allowed by the U.S. Environmental Protection Agency in drinking water does not protect against adverse health effects, particularly in children. Even the so-called Secondary MCL of 2 mg/L proved inadequate [6]. Evidence from modern Pakistan confirms that even this modest level of fluoride in drinking water can have toxic effects in children. In those seasonally hot and arid environments, a greater water consumption necessary to prevent dehydration and the use of fluoridated water in irrigating crops and food preparation, as well as malnutrition can significantly elevate the fluoride intake, exacerbating its adverse physiological effects [28], [29]. In addition, NRC model predictions show that bone fluoride concentrations resulting from lifetime exposure to fluoride in drinking water at 2 or 4 mg/L fall within or exceed the ranges associated with stage II and stage III of skeletal fluorosis, and may increase the risk of overall fractures [7], [26]. Furthermore, the NRC report concluded that fluoride could start or promote cancer, and osteosarcoma is of particular concern, together with other types of bone cancer. A recent large hospital-based case-control study of age-specific fluoride exposure in drinking water and the incidence of osteosarcoma in the United States found a seven-fold increase risk of bone cancer in young boys due to fluorosilicates [67], the most widely used form of fluoride added to drinking water.

### Conclusions

Our findings on the pathologic skeletal and dental features of the ancient residents of *Herculaneum* now indicate that fluorosis was endemic already during Roman times. This evidence and currently available epidemiologic data show a permanent fluoride health hazard for the population living around Vesuvius. At present, the major public health and socio-economic impact of this hazard is underestimated. For several years, the local authorities have allowed a maximum fluoride content of 2.5 mg/L in tap water throughout the area, a value that exceeds WHO as well as national guidelines.

Effects on the skeleton are the best indicators of the toxic responses to fluoride and are considered to have direct public health relevance. According to WHO recommendations, in areas with high fluoride levels and warm climate it would be appropriate to lower the maximum value of 1.5 mg/L established for naturally occurring fluoride in drinking water. Therefore in setting guidelines for fluoride in the densely populated Vesuvius area, the following predisposing factors for fluorosis should be better evaluated: ambient temperature, volume of water intake, other trace elements in the water, a diet based on beverages and food preparation in naturally fluoridated boiled water, and water storage methods. Bearing in mind that progressively higher fluoride intakes lead to increasing risks of dental and skeletal fluorosis, the adoption of low-cost defluoridation methods should be seriously considered and encouraged.

In evaluating all the possible health consequences of exposure to fluoride concentrations higher than the established WHO parameters, it should also be taken into account that the maximum fluoride content of 5.0 mg/L accepted in natural mineral waters protects only the population over 15 years old and only if there is no exposure to fluoride from other sources, as experienced by communities living in volcanic and other fluoride hazard areas.

## Supporting Information

Table S1 **Assessment of ligaments and tendons calcification and ankylosis in the postcranial skeleton of specimens aged 1 to 52-years-old.** 91.8% of the individuals show ossification processes in at least one of the long or flat bones (femur, tibia, clavicle, pelvis), with clavicle the most involved bone (88.2%). Ankylosis, mainly detectable in spine, foot toe distal interphalangeal joint and manubriosternal joint, affects at least one of these three anatomical sites in 39.2% of the individuals.
(DOC)

Table S2 **Occurrence of osteophytosis and spondyloarthritis in spine joints of specimens aged ≥15-years-old.** Osteophytic lesions (moderate+severe+ankylosis) increase towards lumbar joints (17.5% cervical, 26.5% thoracic, 38.8% lumbar), with 27.6% overall occurrence. Spondyloarthritic lesions (moderate+severe+ankylosis) occur in 18.5% of the joints, with lumbar vertebrae the most affected (22.8%).
(DOC)

## References

[1] D'Alessandro W, Kungolos AG, Brebbia CA, Samaras CP, Popov V (2006). Transactions on Biomedicine and Health: Environmental toxicology.

[2] Bocanegra EM, Hernández MA, Usunoff E (2005). Groundwater and human development.

[3] WHO (2008). Guidelines for drinking-water quality. Volume 1- Recommendations.

[4] Littleton J (1999). Paleopathology of skeletal fluorosis.. Am J Phys Anthropol.

[5] Ozsvath DL (2009). Fluoride and environmental health: a review.. Rev Environ Sci Biotechnol.

[6] Ayoob S, Gupta AK (2006). Fluoride in drinking water: a review on the status and stress effects.. Crit Rev Environ Sci Technol.

[7] U.S. NRC (2006). Fluoride in drinking water: A scientific review of EPA's standards.

[8] Heikens A, Sumarti S, van Bergen M, Widianarko B, Fokkert L (2005). The impact of the hyperacid Ijen Crater Lake: risks of excess fluoride to human health.. Sci Tot Environ.

[9] Mastrolorenzo G, Petrone P, Pappalardo L, Sheridan MF (2006). The Avellino 3780-yr-B.P. catastrophe as a worst-case scenario for a future eruption at Vesuvius.. Proc Natl Acad Sci USA.

[10] U.S. NRC (1993). Health effects of ingested fluoride.

[11] Joseph S, Gadhia PK (2000). Sister chromatid exchange frequency and chromosome aberrations in residents of fluoride endemic regions of South Gujarat.. Fluoride.

[12] Thylstrup A, Fejerskov O (1978). Clinical appearance of dental fluorosis in permanent teeth in relation to histologic changes.. Community Dent Oral Epidemiol.

[13] WHO (1970). Fluoride and human health.

[14] Aoba T, Fejerskov O (2002). Dental Fluorosis: Chemistry and biology.. Crit Rev Oral Biol Med.

[15] Skinner MF, Goodman AH, Saunders SR, Katzenberg MA (1992). Anthropological uses of developmental defects of enamel,. Skeletal biology of past peoples: research methods.

[16] Goodman AH, Armelagos GJ (1985). Factors affecting the distribution of enamel hypoplasia within the human permanent dentition.. Am J Phys Anthropol.

[17] Teotia M, Teotia SPS, Singh KP (1998). Endemic chronic fluoride toxicity and dietary calcium deficiency interaction syndromes of metabolic bone disease and deformities in India: Year 2000.. Indian J Pediatr.

[18] Krishnamachari KA (1986). Skeletal fluorosis in humans. A review of recent progress in the understanding of the disease.. Prog Food Nutr Sci.

[19] Resnick D, Niwayama G (1983). Entheses and enthesopathy. Anatomical, pathological, and radiological correlation.. Radiology.

[20] Gupta SK, Gambhir S, Mithal A, Das BK (1993). Skeletal scintigraphic findings in endemic skeletal fluorosis.. Nucl Med Commun.

[21] Weidmann SM, Weatherell JA, Jackson D (1963). The effect of fluoride on bone.. Proceed Nutrit Soc.

[22] Reddy DR (2009). Neurology of endemic skeletal fluorosis.. Neurol India.

[23] Chavassieux P, Seeman E, Delmas PD (2007). Insights into material and structural basis of bone fragility from diseases associated with fractures: how determinants of the biomechanical properties of bone are compromised by disease.. End Rew.

[24] Boivin G, Chavassieux P, Chapuy MC, Baud CA, Meunier PJ (1989). Skeletal fluorosis: histomorphometric analysis of bone changes and bone fluoride in 29 patients.. Bone.

[25] Aggarwal ND (1973). Structure of human fluorotic bone.. J Bone Joint Surg Am.

[26] Li Y, Liang C, Slemenda CW, Ji R, Sun S (2001). Effect of long-term exposure to fluoride in drinking water on risks of bone fractures.. J Bone Min Res.

[27] Ortner DJ (2003). Identification of pathological conditions in human skeletal remains.

[28] Lukacs JR (1984). Dental fluorosis in early Neolithic Pakistan.. Am J Phys Anthropol.

[29] Lukacs JR, Retief DH, Jarrige J-F (1985). Dental disease in prehistoric Baluchistan.. Nat Geo Res.

[30] Yoshimura K, Nakahashi T, Saito K (2006). Why did the ancient inhabitants of Palmyra suffer fluorosis?. J Archaeol Sci.

[31] Mastrolorenzo G, Petrone PP, Pagano M, Incoronato A, Baxter PJ (2001). Herculaneum victims of Vesuvius in AD 79.. Nature.

[32] Mastrolorenzo G, Petrone P, Pappalardo L, Guarino FM (2010). Lethal thermal impact at Periphery of Pyroclastic Surges: Evidences at Pompeii.. PLoS ONE.

[33] Capasso L (2001). I fuggiaschi di Ercolano. Paleobiologia delle vittime dell'eruzione vesuviana del 79 d.C..

[34] Hedges REM (2002). Bone diagenesis: an overview of processes.. Archaeometry.

[35] Tressaud A (2006). Fluorine and the environment, agrochemicals, archaeology, green chemistry and water.

[36] Buikstra JE, Ubelaker DH (1994). Standards for Data Collection from Human Skeletal Remains.

[37] Hillson S (2005). Teeth.

[38] Dean HT, Moulton FR (1942). The investigation of physiological effects by the epidemiological method,. Fluorine and dental health.

[39] Rogers J, Shepstone L, Dieppe P (1997). Bone formers: osteophyte and enthesophyte formation are positively associated.. Ann Rheum Dis.

[40] Jurmain RD (1990). Paleoepidemiology of a central California prehistoric population from CA-ALA-329: II. Degenerative disease.. Am J Phys Anthropol.

[41] Jurmain RD (1977). Stress and etiology of osteoarthritis.. Am J Phys Anthropol.

[42] Guarino FM, Angelini F, Vollono C, Orefice C (2006). Bone preservation in human remains from the Terme del Sarno at Pompeii using light microscopy and scanning electron microscopy.. J Archaeol Sci.

[43] Cheng TP, Anderson HD, Mills DS, Spate VL, Baskett CK (1997). Determination of the fluoride distribution in rabbit bone using instrumental neutron activation analysis.. J Radioanal Nucl Chem.

[44] Wittmers LE, Aufderheide AC, Pounds JG, Jones KV, Angel JL (2008). Problems in determination of skeletal lead burden in archaeological samples: an example from the first African Baptist Church population.. Am J Phys Anthropol.

[45] Schurr MR (1989). Fluoride dating of prehistoric bones by ion selective electrode.. J Archeol Sci.

[46] Capasso L, Kennedy KAR, Wilczak CA (1999). Atlas of occupational markers of human skeletal remains.

[47] Haettich B, Lebreton C, Prier A, Kaplan G (1991). Magnetic resonance imaging of fluorosis and stress fractures due to fluoride.. Rev Rhum Mal Osteoartic.

[48] Feemster Jashemski W, Meyer FG (2002). The natural history of Pompeii.

[49] Bisel C (1991). The human skeletons of Herculaneum.. Int J Anthropol.

[50] Eble DM, Deaton TG, Wilson FC, Bawden JW (1992). Fluoride concentrations in human and rat bone.. J Pub Health Dent.

[51] Sastri CS, Iyengar V, Blondiaux G, Tessier Y, Petri H (2001). Fluorine determination in human and animal bones by particle-induced gamma-ray emission.. Fresenius J Anal Chem.

[52] WHO (2002). Environmental health criteria 227: Fluorides.

[53] Choubisa SL (2001). Endemic fluorosis in southern Rajasthan, India.. Fluoride.

[54] Vargiu R, Bellini GR, Mancinelli D, Santoro P, Miranda G (2007). Lazio e Sabina. Scoperte, scavi e ricerche.

[55] Robb J, Bigazzi R, Lazzarini L, Scarsini C, Sonego F (2001). Social “status” and “biological” status: a comparison of grave goods and skeletal indicators from Pontecagnano.. Am J Phys Anthropol.

[56] Sperduti A (1997). Life conditions of a Roman Imperial Age population: Occupational stress markers and working activities in Lucus Feroniae (Rome, lst-2nd cent. AD).. Hum Evol.

[57] Capasso L (1998). Work-related syndesmoses on the bones of children who died at Herculaneum.. The Lancet.

[58] Manzi G, Salvadei L, Vienna A, Passarello P (1999). Discontinuity of life conditions at the transition from the Roman Imperial Age to the Early Middle Ages: Example from Central Italy evaluated by pathological dento-alveolar lesions.. Am J Hum Biol.

[59] Cucina A, Vargiu R, Mancinelli D, Ricci R, Santandrea A (2006). The necropolis of Vallerano (Rome, 2nd–3rd century AD): an anthropological perspective on the ancient Romans in the Suburbium.. Int J Osteoarchaeol.

[60] Wondwossen F, Nordrehaug Åstrøm A, Kjell Bjorvatn K, Bårdsen A (2004). The relationship between dental caries and dental fluorosis in areas with moderate- and high-fluoride drinking water in Ethiopia.. Community Dent Oral Epidemiol.

[61] U.S. Environmental Protection Agency (2002). National Primary Drinking Water Regulations, Drinking Water Contaminants, Fluoride.

[62] Connet M (2004). Fluoride & Bone Damage: Published Data.

[63] Grimaldo M, Turrubiartes F, Milan J, Pozos A, Alfaro C (1995). Endemic fluorosis in San Luis Potosi, Mexico. I. Identification of risk factors associated with human exposure to fluoride.. Environ Res.

[64] Jolly SS, Singh BM, Mathur OC, Malhotra KC (1968). Epidemiological, clinical, and biochemical study of endemic dental and skeletal fluorosis in Punjab.. Brit Med J.

[65] Gombos F, Mangoni Di Stefano C, Ruggiero M (1994). Investigation into fluorosis in school children of two Vesuvian towns.. Arch Stomat.

[66] Regione Campania (2008). Deliberazione N. 2095 del 31/12/2008.. Deroga al valore massimo ammissibile del parametro fluoro contenuto nelle acque al consumo umano nei Comuni del comprensorio vesuviano per l'anno 2009.

[67] Bassin EB, Wypij D, Davis RB, Mittleman MA (2006). Age-specific fluoride exposure in drinking water and osteosarcoma (United States).. Cancer Causes Control.

