# Supplementary material for: Enduring Fluoride Health Hazard for the Vesuvius Area Population: The Case of AD 79 Herculaneum
Source: PLoS One. 2011 Jun 16;6(6):e21085. doi: 10.1371/journal.pone.0021085 (PMC3116870; doi:10.1371/journal.pone.0021085)
Supplement: Table S1 — Assessment of ligaments and tendons calcification and ankylosis in the postcranial skeleton of specimens aged 1 to 52-years-old. 91.8% of the individuals show ossification processes in at least one of the long or flat bones (femur, tibia, clavicle, pelvis), with clavicle the most involved bone (88.2%). Ankylosis, mainly detectable in spine, foot toe distal interphalangeal joint and manubriosternal joint, affects at least one of these three anatomical sites in 39.2% of the individuals. (DOC) [file pone.0021085.s001.doc]

**Table S1**. Assessment of ligaments and tendons calcification and ankylosis in the postcranial skeleton of specimens aged 1 to 52-years-old

| **N** | **Ind.** | **Sex** | **Mean age** | **Femur** | **Tibia** | **Clavicle** | **Ileum** | **Ischium** | **Total** | **Toes ankylosis** | **Spine ankylosis** | **M-sternal ankylosis** | **Total** |
| --- | --- | --- | --- | --- | --- | --- | --- | --- | --- | --- | --- | --- | --- |
| 1 | 5:1 | M | 14.5 | P | A | P | P | P | P | / | A | / | A |
| 2 | 5:2 | F | 20.2 | A | A | P | P | P | P | / | A | A | A |
| 3 | 5:3 | M | 20.2 | P | P | P | P | P | P | / | A | P | P |
| 4 | 10:1 | M | 39.0 | P | P | P | P | P | P | A | A | / | A |
| 5 | 10:2 | M | 15.0 | P | P | P | A | A | P | A | / | / | A |
| 6 | 10:3 | M | 50.0 | P | P | P | P | P | P | P | P Tho/Sa-Il | A | P |
| 7 | 10:4 | F | 29.2 | A | P | P | P | P | P | A | A | / | A |
| 8 | 10:5 | M | 21.4 | P | P | P | A | P | P | / | A | A | A |
| 9 | 10:6 | M | 30.8 | A | A | P | A | A | P | A | A | P | P |
| 10 | 10:7 | M | 35.5 | A | P | P | P | P | P | P | A | A | P |
| 11 | 10:8 | M | 13.2 | A | A | A | A | A | A | A | / | / | A |
| 12 | 10:9 | M | 14.7 | A | A | A | A | A | A | A | / | / | A |
| 13 | 10:10 | M | 35.1 | A | A | P | A | A | P | A | A | A | A |
| 14 | 10:11A | F | 31.3 | P | P | P | A | A | P | A | A | A | A |
| 15 | 10:11B | M | 34.3 | / | / | / | / | / | / | / | / | / | / |
| 16 | 10:12 | M | 34.0 | P | P | P | P | P | P | P | A | P | P |
| 17 | 10:13 | M | 33.9 | P | P | P | P | P | P | A | P Tho/Lu | A | P |
| 18 | 10:14 | M | 37.0 | P | P | P | P | P | P | / | A | P | P |
| 19 | 10:15 | F | 28.6 | P | A | P | P | P | P | A | A | A | A |
| 20 | 10:16 | F | 36.3 | P | P | P | P | P | P | A | P Lu | A | P |
| 21 | 10:17 | M | 35.5 | A | A | P | P | P | P | / | A | A | A |
| 22 | 10:18 | F | 38.0 | A | A | P | P | P | P | P | P Sa-Lu | A | P |
| 23 | 10:19 | M | 30.0 | A | A | P | A | A | P | A | A | A | A |
| 24 | 10:20 | M | 44.0 | P | P | P | P | P | P | A | A | A | A |
| 25 | 10:21 | M | 37.5 | P | P | P | / | P | P | A | A | A | A |
| 26 | 10:22 | M | 20.5 | A | P | P | A | A | P | A | A | / | A |
| 27 | 10:23 | M | 36.5 | P | P | A | A | P | P | / | A | A | A |
| 28 | 10:24 | F | 41.1 | P | P | P | P | P | P | / | P Sa-Il | / | P |
| 29 | 10:25 | M? | 21.5 | P | P | / | / | / | P | P | / | / | P |
| 30 | 10:25B | ? | 21.5 | / | P | / | / | / | P | / | / | / | / |
| 31 | 10:26 | M | 9.2 | P | P | P | / | / | P | / | / | / | / |
| 32 | 10:27 | F | 5.3 | / | / | / | / | / | / | / | / | / | / |
| 33 | 10:28 | F | 36.5 | P | A | P | P | P | P | A | A | A | A |
| 34 | 10:29 | F | 29.0 | P | A | P | / | P | P | P | A | A | P |
| 35 | 10:30 | M? | 2.5 | / | / | / | / | / | / | / | / | / | / |
| 36 | 10:32 | M | 9.5 | A | P | P | / | / | P | / | / | / | / |
| 37 | 10:33 | F | 12.0 | P | P | / | / | / | P | / | / | / | / |
| 38 | 10:34 | F? | 19.5 | A | A | / | / | P | P | A | / | / | A |
| 39 | 10:35 | M | 24.5 | / | P | / | / | / | P | A | / | / | A |
| 40 | 10:36 | M | 16.0 | P | P | A | A | A | P | A | / | / | A |
| 41 | 10:38 | ? | 13.5 | / | / | / | / | / | / | / | / | / | / |
| 42 | 10:39 | M | 13.5 | / | / | / | / | / | / | / | / | / | / |
| 43 | 10:40 | ? | 12.5 | / | / | / | / | / | / | / | / | / | / |
| 44 | 10:41 | F | 1.0 | / | / | / | / | / | / | / | / | / | / |
| 45 | 12:1 | M? | 4.0 | / | / | / | / | / | / | / | / | / |  |
| 46 | 12:2 | F | 26.0 | P | A | A | P | P | P | / | A | / | A |
| 47 | 12:3 | F | 27.6 | A | A | / | A | A | A | / | A | / | A |
| 48 | 12:4 | M | 27.0 | P | P | / | P | P | P | / | A | / | A |
| 49 | 12:5 | M? | 16.6 | A | A | / | / | A | A | / | / | / | / |
| 50 | 12:6 | F? | 8.5 | / | / | / | / | / | / | / | / | / | / |
| 51 | 12:7 | M? | 17.3 | A | A | / | A | A | A | / | A | / | A |
| 52 | 12:8 | M? | 37.8 | A | A | P | A | P | P | P | A | / | P |
| 53 | 12:9 | F | 25.0 | P | P | P | P | P | P | P | A | A | P |
| 54 | 12:10 | M | 9.0 | P | P | P | / | / | P | / | / | / | / |
| 55 | 12:11 | M | 52.3 | P | P | P | P | P | P | / | P Tho/Sa-Lu | P | P |
| 56 | 12:12 | M | 3.0 | / | / | / | / | / | / | / | / | / | / |
| 57 | 12:13 | F? | 37.3 | P | P | P | A | P | P | A | A | A | A |
| 58 | 12:14 | M | 12.0 | P | P | P | / | / | P | / | / | / | / |
| 59 | 12:15 | F | 32.7 | P | P | P | P | P | P | A | A | A | A |
| 60 | 12:16 | M | 37.2 | P | P | P | P | / | P | P | P Tho | A | P |
| 61 | 12:17 | ? | 5.5 | / | / | / | / | / | / | / | / | / | / |
| 62 | 12:18 | F? | 3.5 | / | / | / | / | / | / | / | / | / | / |
| 63 | 12:19 | M | 33.9 | A | A | P | P | P | P | A | A | A | A |
| 64 | 12:20 | M | 18.0 | A | A | P | A | A | P | A | / | / | A |
| 65 | 12:21 | F | 32.1 | P | P | / | P | P | P | P | A | / | P |
| 66 | 12:22 | M | 20.5 | A | A | P | A | A | P | / | / | / | / |
| 67 | 12:23 | M | 42.0 | P | P | P | P | P | P | A | P Tho/Lu | P | P |
| 68 | 12:24 | M | 9.5 | / | / | P | / | / | P | / | / | / | / |
| 69 | 12:25 | M | 10.5 | P | P | P | / | / | P | / | / | / | / |
| 70 | 12:26 | M | 31.0 | P | P | P | P | P | P | / | A | P | P |
| 71 | 12:27 | M | 36.0 | P | P | P | P | P | P | A | P Tho | A | P |
| 72 | 12:28 | F | 36.0 | A | P | A | P | P | P | / | A | A | A |
| 73 | 12:29 | F? | 12.0 | / | / | / | / | / | / | / | / | / | / |
| 74 | 12:30 | F | 35.2 | P | / | P | P | P | P | A | A | A | A |
| 75 | 12:31 | F? | 29.9 | / | / | / | / | / | / | / | / | / | / |
|  |  |  | **N** | 37/58 | 38/59 | 45/51 | 31/48 | 36/50 | 56/61 | 10/36 | 9/43 | 7/32 | 20/51 |
|  |  |  | ***%*** | *63.8* | *64.4* | *88.2* | *64.6* | *72.6* | ***91.8*** | *27.8* | *20.9* | *21.9* | ***39.2*** |

Ind. = specimen; P = presence; A = absence; / = unreliable; M-sternal = manubriosternal; Tho = thoracic vertebrae;

Lu = lumbar vertebrae; Sa-Il = sacroiliac; Sa-Lu = sacrolumbar
