# Supplementary material for: Enduring Fluoride Health Hazard for the Vesuvius Area Population: The Case of AD 79 Herculaneum
Source: PLoS One. 2011 Jun 16;6(6):e21085. doi: 10.1371/journal.pone.0021085 (PMC3116870; doi:10.1371/journal.pone.0021085)
Supplement: Table S2 — Occurrence of osteophytosis and spondyloarthritis in spine joints of specimens aged ≥15-years-old. Osteophytic lesions (moderate+severe+ankylosis) increase towards lumbar joints (17.5% cervical, 26.5% thoracic, 38.8% lumbar), with 27.6% overall occurrence. Spondyloarthritic lesions (moderate+severe+ankylosis) occur in 18.5% of the joints, with lumbar vertebrae the most affected (22.8%). (DOC) [file pone.0021085.s002.doc]

**Table S2**. Occurrence of osteophytosis and spondyloarthritis in spine joints of specimens aged ≥ 15-years-old

| **Osteophytosis** | **Cervical** | | | **Thoracic** | | | **Lumbar** | | | **Ce + Tho + Lu** | | |
| --- | --- | --- | --- | --- | --- | --- | --- | --- | --- | --- | --- | --- |
|  | ♂ | ♀ | *%* | ♂ | ♀ | *%* | ♂ | ♀ | *%* | ♂ | ♀ | *%* |
| Absent | 110 | 45 | *79.9* | 199 | 93 | *67.9* | 85 | 39 | *56.6* | 394 | 177 | *67.7* |
| Traces | 2 | 3 | *2.6* | 12 | 12 | *5.6* | 4 | 6 | *4.6* | 18 | 21 | *4.6* |
| A + T *%* | *80.6* | *87.3* | *82.5* | *73.5* | *73.4* | *73.5* | *66.4* | *52.9* | *61.2* | *73.6* | *70.0* | *72.4* |
| Moderate | 15 | 4 | *9.8* | 31 | 23 | *12.5* | 17 | 21 | *17.4* | 63 | 48 | *13.2* |
| Severe | 11 | 3 | *7.2* | 31 | 15 | *10.7* | 25 | 16 | *18.7* | 67 | 34 | *12.0* |
| Ankilosys | 1 | 0 | *0.5* | 14 | 0 | *3.3* | 3 | 3 | *2.7* | 18 | 3 | *2.5* |
| M + S + A *%*  *by total count* | *13.9* | *3.6* | *17.5* | *17.7* | *8.8* | *26.5* | *20.6* | *18.3* | *38.8* | *17.6* | *10.1* | *27.6* |
| M + S + A *%*  *by gender* | *19.4* | *12.7* | *17.5* | *26.5* | *26.6* | *26.5* | *33.6* | *47.1* | *38.8* | *26.4* | *30.0* | *27.6* |
| S + A *%*  *by total count* | *6.2* | *1.6* | *7.7* | *10.5* | *3.5* | *14.0* | *12.8* | *8.7* | *21.5* | *10.1* | *4.4* | *14.5* |
| S + A *%*  *by gender* | *8.6* | *5.4* | *7.7* | *15.7* | *10.5* | *14.0* | *20.9* | *22.4* | *21.5* | *15.2* | *13.1* | *14.5* |
| *total %* | *100.0* | *100.0* | *100.0* | *100.0* | *100.0* | *100.0* | *100.0* | *100.0* | *100.0* | *100.0* | *100.0* | *100.0* |
| N (joints) | 139 | 55 | 194 | 287 | 143 | 430 | 134 | 85 | 219 | 560 | 283 | 843 |

| **Spondyloarthritis** | **Cervical** | | | | **Thoracic** | | | **Lumbar** | | | **Ce + Tho + Lu** | | |
| --- | --- | --- | --- | --- | --- | --- | --- | --- | --- | --- | --- | --- | --- |
|  | ♂ | ♀ | | *%* | ♂ | ♀ | *%* | ♂ | ♀ | *%* | ♂ | ♀ | *%* |
| Absent | 130 | 48 | | *69.0* | 208 | 114 | *29.7* | 103 | 37 | *37.4* | 441 | 199 | *71.5* |
| Traces | 25 | 10 | | *13.6* | 15 | 16 | *7.3* | 9 | 14 | *10.9* | 49 | 40 | *10.0* |
| A+T% | *82.9* | *81.7* | | *82.6* | *81.7* | *85.0* | *82.9* | *85.5* | *63.8* | *77.2* | 82.9 | 78.6 | *81.5* |
| Moderate | 28 | 11 | | *15.1* | 29 | 18 | *11.0* | 10 | 25 | *16.6* | 67 | 54 | *13.5* |
| Severe | 4 | 2 | | *2.3* | 21 | 5 | *6.1* | 9 | 4 | *6.2* | 34 | 11 | *5.0* |
| Ankilosys | 0 | 0 | | *0.0* | 0 | 0 | *0.0* | 0 | 0 | *0.0* | 0 | 0 | *0.0* |
| M + S + A *%*  *by total count* | *12.3* | *5.1* | | *17.4* | *11.7* | *5.4* | *17.1* | *9.0* | *13.8* | *22.8* | *11.3* | *7.2* | *18.5* |
| M + S+ A *%*  *by gender* | *17.1* | *18.3* | | *17.4* | *18.3* | *15.0* | *17.1* | *14.5* | *36.2* | *22.8* | *17.1* | *21.4* | *18.5* |
| S + A *%*  *by total count* | *1.6* | *0.8* | | *2.3* | *4.9* | *1.2* | *6.1* | *4.3* | *1.9* | *6.2* | *3.8* | *1.2* | *5.0* |
| S + A *%*  *by gender* | *2.1* | *2.8* | | *2.3* | *7.7* | *3.3* | *6.1* | *6.9* | *5.0* | *6.2* | *5.8* | *3.6* | *5.0* |
| *total %* | *100.0* | *100.0* | | *100.0* | *100.0* | *100.0* | *100.0* | *100.0* | *100.0* | *100.0* | *100.0* | *100.0* | *100.0* |
| N (joints) | 187 | | 71 | 258 | 273 | 153 | 426 | 131 | 80 | 211 | 591 | 304 | 895 |

N = number of articulations; ♂ = number of male articulations; ♀ = number of female articulations
